# A1-reprogrammed mesenchymal stromal cells prime potent antitumoral responses

**DOI:** 10.1016/j.isci.2024.109248

**Published:** 2024-02-17

**Authors:** Marina Pereira Gonçalves, Roudy Farah, Jean-Pierre Bikorimana, Jamilah Abusarah, Nehme EL-Hachem, Wael Saad, Sebastien Talbot, Daniela Stanga, Simon Beaudoin, Sebastien Plouffe, Moutih Rafei

**Affiliations:** 1 Molecular Biology Program, Université de Montréal, Montreal, QC, Canada; 2 Department of Microbiology, Infectious Diseases and Immunology, Université de Montréal, Montreal, QC, Canada; 3 Department of Pharmacology and Physiology, Université de Montréal, Montreal, QC, Canada; 4 Pediatric Hematology-Oncology Division, Centre Hospitalier Universitaire Sainte-Justine Research Centre, Montreal, QC, Canada; 5 Department of Biomedical and Molecular Sciences, Queen’s University, Kingston, ON, Canada; 6 Defence Therapeutics Inc., Research and Development branch, Montreal, QC, Canada

**Keywords:** Classification Description, Immunology, Pharmaceutical engineering, Cancer

## Abstract

Mesenchymal stromal cells (MSCs) have been modified via genetic or pharmacological engineering into potent antigen-presenting cells-like capable of priming responding CD8 T cells. In this study, our screening of a variant library of Accum molecule revealed a molecule (A1) capable of eliciting antigen cross-presentation properties in MSCs. A1-reprogrammed MSCs (ARM) exhibited improved soluble antigen uptake and processing. Our comprehensive analysis, encompassing cross-presentation assays and molecular profiling, among other cellular investigations, elucidated A1’s impact on endosomal escape, reactive oxygen species production, and cytokine secretion. By evaluating ARM-based cellular vaccine in mouse models of lymphoma and melanoma, we observe significant therapeutic potency, particularly in allogeneic setting and in combination with anti-PD-1 immune checkpoint inhibitor. Overall, this study introduces a strong target for developing an antigen-adaptable vaccination platform, capable of synergizing with immune checkpoint blockers to trigger tumor regression, supporting further investigation of ARMs as an effective and versatile anti-cancer vaccine.

## Highlights

• Treatment with A1/antigen mix reprograms MSCs into antigen-presenting cells
• The antigen cross-presenting ability of ARM cells require ROS and UPR
• ARMs synergize with immune-checkpoint inhibitors in priming potent antitumoral activity

## Graphical abstract

**Figure:**
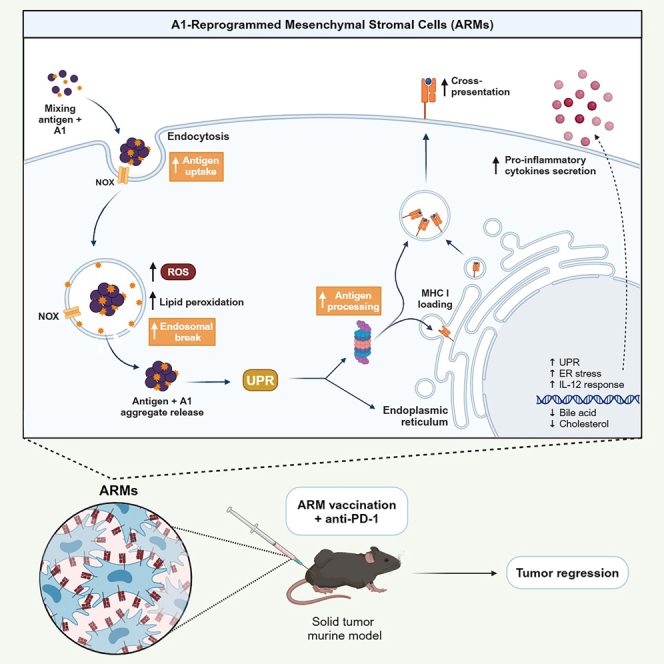

## Introduction

Cancer vaccines offer promising alternatives to current immunotherapy strategies as they can potentially cure established tumors while imprinting a long-lasting response through immunological memory.^1^ In addition, cancer vaccines can help boosting pre-existing effector cells while priming a fresh cohort of tumor-reactive T cells^2^ if designed to target a broader range of antigens.^3^^,^^4^ Different types of cancer vaccines can be categorized by antigen type (predefined or anonymous) or delivery method (direct or via the use of antigen-presenting cells [APCs]).^2^ Several studies suggest that antigen delivery by APCs, such as dendritic cells (DCs), offers greater effectiveness compared to whole tumor cells administration.^2^^,^^5^ These observations, combined with the fact that DCs are professional APCs, make them a logical choice for cancer vaccination.^6^ However, the natural bloodstream scarcity of DCs combined with the hurdles associated with the use of *ex vivo* generated monocyte-derived (Mo)-DCs pose several limitations to the effectiveness of DC-based cancer vaccines.^7^^,^^8^

To address these concerns, we have previously explored mesenchymal stromal cells (MSCs) as an alternative vaccine platform.^9^^,^^10^^,^^11^^,^^12^ MSCs are versatile, can be acquired from various sources, and are highly proliferative in cell culture.^13^ Their isolation is considerably simple and cost-effective,^14^^,^^15^ and their safety has been extensively demonstrated in clinical studies.^16^ Although their inherent immunosuppressive and regenerative profiles make them an ideal treatment modality for the induction of tolerance, graft survival, suppression of immune-based disorders, and regenerative medicine,^17^ MSCs harbor a unique plasticity allowing them to acquire a pro-inflammatory phenotype under certain stimuli.^18^^,^^19^^,^^20^ The latter characteristic makes them potential candidates for cell-based vaccines. For instance, our group has previously demonstrated how MSCs can be genetically engineered or pharmacologically reprogrammed to behave as APCs capable of cross-presenting antigens resulting in tumor control.^9^^,^^11^^,^^12^

Cross-presentation is an indispensable process for antitumoral immunity as it is the sole mechanism by which exogenous antigens (like those shredded by cancer cells) can be processed and presented through major histocompatibility complex (MHC) class I molecules.^21^ A crucial process in cross-presentation is antigen export from endosomes to the cytosol.^22^^,^^23^^,^^24^ This step may involve the activation of NADPH oxidases (NOX),^25^^,^^26^ which generate intra-endosomal reactive oxygen species (ROS), helping to create an alkaline environment.^25^^,^^26^^,^^27^ Such alkalization prevents endosomal maturation and unspecific antigen degradation that could occur upon lysosomal protease digestion. In addition, ROS trigger lipid peroxidation leading to endosomal membrane disruption.^24^^,^^28^ As a result, intact antigens can reach the cytosol enabling more efficient processing by the proteasomal machinery to generate a larger pool of immunogenic peptides to be presented on the cell surface.^29^ We thus focused on endosomal antigen export as a primary target in our endeavor to pharmacologically convert MSCs into potent APCs. To do so, we explored the use of Accum, a technology initially designed to enhance intracellular drug delivery and accumulation by disrupting endosomal membranes.^30^^,^^31^

To further advance the results obtained from previous studies on cancer vaccine development, we opted to recruit and benefit from the properties of MSCs and Accum combination. Since the Accum structure can be modulated and further optimized, we generated a variant library of Accum and screened it to identify molecules capable of triggering unique APC-like behavior in MSCs. We herein present an Accum variant (named A1) capable of reprogramming MSCs into powerful antigen cross-presenting cells (ARMs). At the molecular level, A1 causes multiple events starting with protein aggregation, intra-endosomal ROS production breaking the endosomal membrane allowing protein release to the cytosol, and protein degradation by the proteasome. The net outcome culminates in a potent antitumoral response in mouse models of lymphoma and melanoma.

## Results

### Identification of A1, a molecule inducing cross-presentation in MSCs

**Figure 1:**
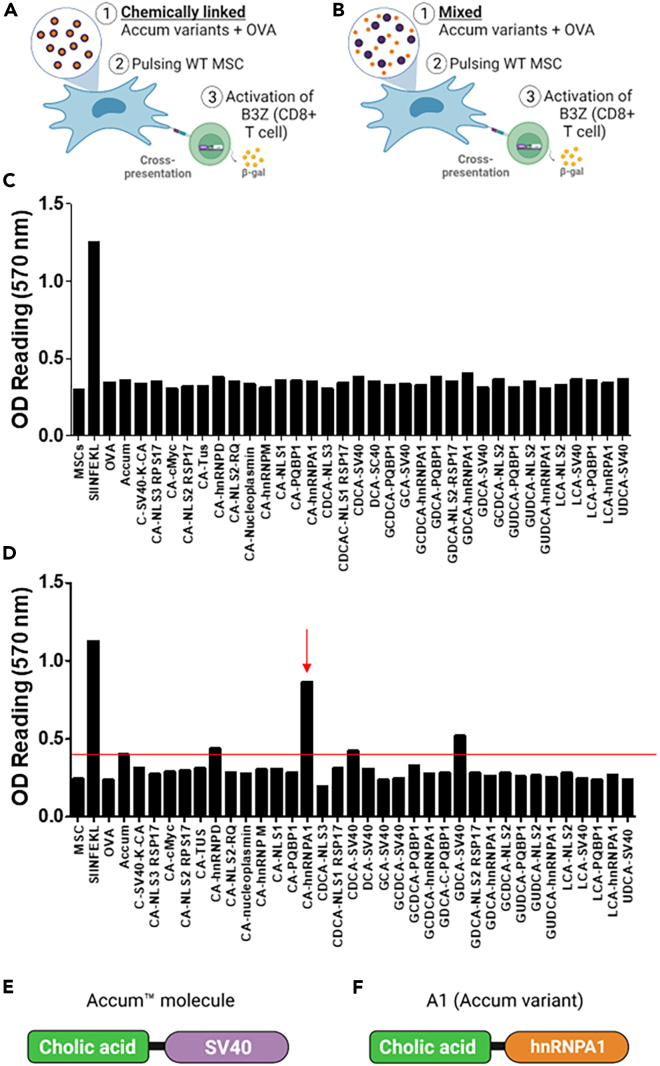
Screening Accum variants capable of inducing cross-presentation in MSCs (A) Schematic diagram of the antigen cross-presentation assay in which MSC are pulsed using different Accum-linked OVA variants. Treated MSCs are co-cultured with B3Z, a T cell hybridoma expressing a TCR recognizing the OVA-derived SIINFEKL peptide in the context of MHC I. B3Z express beta-galactosidase (lacZ) driven by NF-AT elements activated after recognition and activation. (B) Same as in (A), except that the Accum variants were admixed with OVA protein. (C) Antigen presentation assay screening the Accum-linked OVA constructs. SIINFEKL is used as a positive technical control for B3Z, MSCs (untreated), OVA, and Accum are used as negative controls. (D) Same as in (C), except that the antigen presentation assay is screening the Accum variants admixed with OVA. (E) Cartoon structure of the original Accum molecule, comprising cholic acid (CA) as the bile acid module and SV40 as the NLS moiety. (F) Cartoon structure of the A1 Accum variant, which maintains the CA, but linked to the NLS hnRNPA1.

Accum is a technology initially designed to enhance drug delivery and intracellular accumulation when conjugated to biomedicines.^32^ Its unique structure consists of a bile acid linked to a peptide-based nuclear localization signal (NLS). Inspired by the infection mechanism of non-enveloped viruses, the Accum composition destabilizes the endosomal membrane, provoking the escape of captured cargo to the cytosol.^30^^,^^31^ In fact, we previously demonstrated how OVA-linked Accum potentiates the antitumoral effect induced by DCs in the context of cancer vaccination,^31^ which prompted us to assess whether similar effects could be instilled in MSCs. To test this hypothesis, we screened a library of 34 Accum variants (with different bile acids and/or NLS sequences) using two different cross-presentation assays. In the first assay (Figure 1A), the variants were chemically linked to the OVA protein as opposed to the second assay, where the variants were admixed with OVA (Figure 1B). Although no signal could be detected in any of the experimental conditions tested in the chemically linked assay (Figure 1C), one Accum variant (cholic acid-hnRNPA1—hereafter referred to as A1) successfully triggered the activation of B3Z hybridoma in the admixed condition (Figure 1D). B3Z T cell hybridoma cells express a T-cell receptor (TCR) recognizing the OVA-derived SIINFEKL peptide on MHC I, upon recognition of the peptide receptor complex on APC, TCR activation elicits nuclear factor of activated T-cell (NFAT) activity leading to β-galactosidase (LacZ) synthesis. The amount of LacZ produced can then be quantified by the hydrolysis of the chromogenic substrate chlorophenol red-β-D-galactopyranoside (CPRG).^33^ Despite sharing a bile acid similar to other variants, including the original Accum molecule (Figure 1E), our data indicate that the A1 molecule (Figure 1F) exhibits properties that are distinct from all tested variants.

### A1 pulsing enhances antigen uptake and processing while triggering endosomal escape

**Figure 2:**
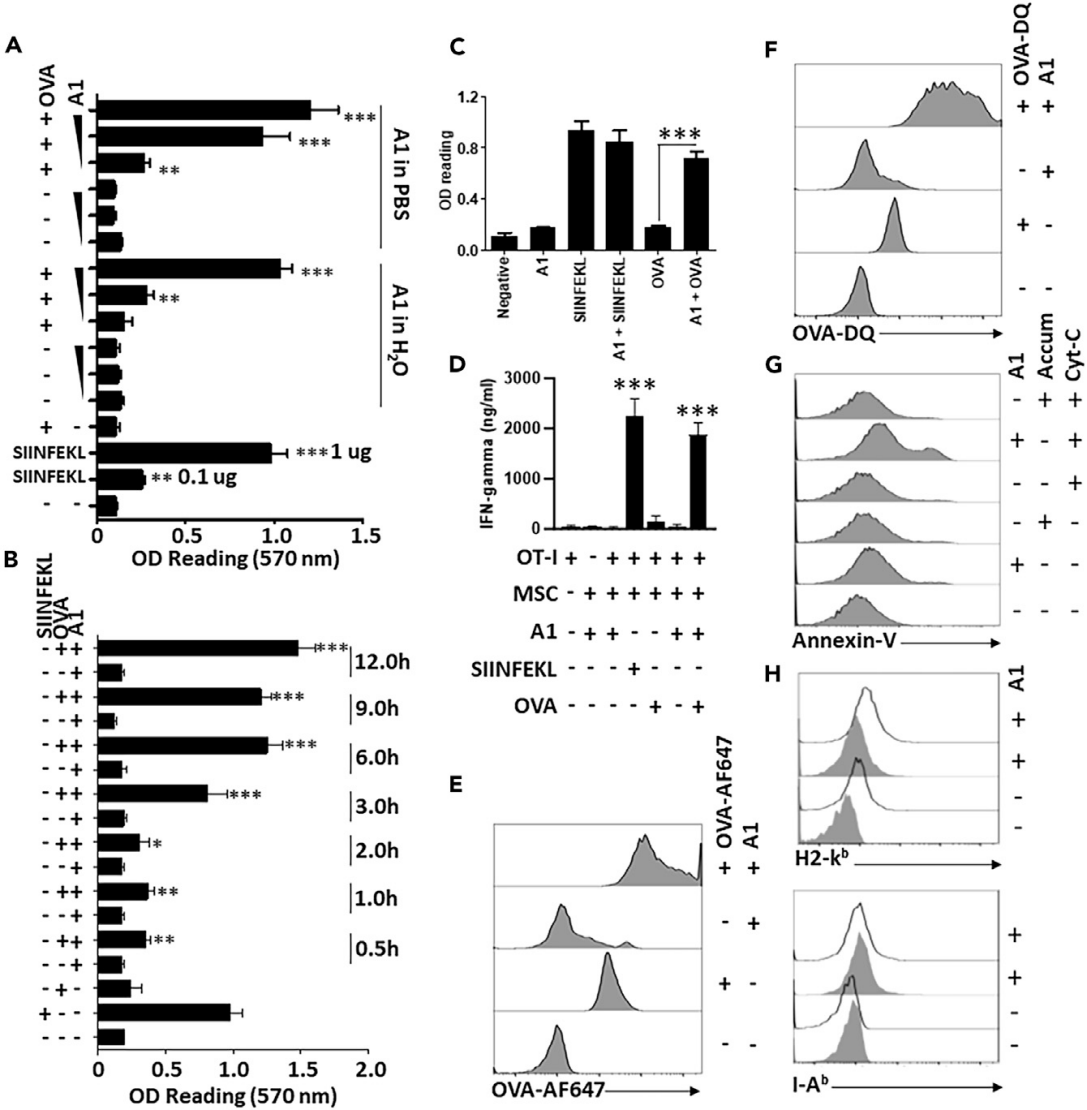
Characterizing the cross-presentation capacity of the A1 variant (A) Antigen cross-presentation assay conducted using A1 diluted in PBS or water to compare the impact of A1 formulations in the capacity to induce cross-presentation. (B) Antigen cross-presentation assay conducted using different pulsing time points to optimize the treatment period. (C) Antigen cross-presentation assay conducted using A1 admixed with SIINFEKL to assess the impact on antigen presentation, and A1 admixed with OVA to confirm induction of cross-presentation. (D) Similar to(C) but using OT-T-derived CD8 T cells as responding lymphocytes. (E) Evaluating the effect of A1 on antigen uptake by MSCs using OVA-AF647. (F) Assessing the effect of A1 on antigen processing by MSCs using OVA-DQ. (G) Assessing the endosomal damaging properties of A1 on MSCs co-treated with Cyt-C. After endocytosis, Cyt-C can induce apoptosis if reaching the cytosol upon endosomal break. Annexin-V staining was used to analyze changes in cell death. (H) Representative flow cytometry analysis of H2-K^b^ (top panel) and I-A^b^ (lower panel). Gray histograms show isotype controls, whereas test-stained samples are in white. For panels A-C, n = 4/group. Asterisks refers to statistical differences between the group labeled by the symbol and the group of untreated MSCs. Results are presented as average mean with standard deviation (S.D.) error bars, and statistical significance is represented with asterisks: ∗*p* ˂ 0.05, ∗∗*p* ˂ 0.01, ∗∗∗*p* ˂ 0.001. See also Figures S1–S3.

Following A1 identification, we next tested various parameters to determine the best condition yielding a balance between maximal antigen cross-presentation and absent/minimal cell death. To do so, we first compared the impact of PBS versus distilled water as diluents for A1. Although the B3Z T cells responded to MSCs treated with both 25 and 50 μM of A1 diluted in PBS (Figure 2A), only the 50 μM dose worked in the water condition, with no apparent cell death induced according to Annexin-V/PI staining on treated MSCs (Figures S1A and S1B). Based on these data, we next determined the optimal pulsing duration to be 6 h (Figure 2B) with 0.5 mg/mL being the minimal and 1 mg/mL being the optimal protein concentration required to obtain a detectable B3Z response (Figure S1C). So far, all conducted tests were centered on antigen cross-presentation with no assessment of A1 potential impact on the process of antigen presentation. As shown in Figure 2C, robust activation of B3Z (confirmed using OT-I cells—Figure 2D) occurred upon exposure to A1+OVA-treated MSCs, when compared with OVA-only pulsed MSCs, while the presence of A1 did not induce significant changes when the cells were pulsed with the SIINFEKL peptide. To investigate the mechanistic basis of A1-induced cross-presentation, we next examined if A1 affects antigen uptake and processing, which are both critical for cross-presentation. When monitored for their capacity to capture fluorescent OVA (OVA-AF647), a significant increase in fluorescence emission was detected in MSCs treated with the antigen/A1 mix compared to control groups (Figure 2E). Similar results were obtained with respect to antigen processing using OVA-DQ, a self-quenching fluorescent probe emitting fluorescence upon proteolysis (Figure 2F). Similar uptake and processing results were obtained when re-tested on human-derived MSCs (Figures S2A–S2D). In line with these observations, an endosomal disruption assay was next conducted to assess whether A1 elicits antigen export from the endosome to the cytosol. The assay involved pulsing MSCs with exogenous Cyt-C and monitoring changes in cell death.^24^ Since Cyt-C is promptly endocytosed and cannot naturally cross the endosomal membrane, its ability to induce apoptosis relies on reaching the cytosol as an intact antigen.^22^ As shown in Figure 2G, Cyt-C only triggers cell death when combined with A1, an effect not observed with the parent Accum molecule. Furthermore, the observed effects did not change the overall H2-K^b^ or I-A^b^ cell surface levels (Figure 2H) nor the innate phenotype of MSCs as shown by CD44, CD45, CD73, and CD90 marker detection by flow cytometry (Figure S3). Overall, our data indicate a central role for A1 on various cross-presentation-related processes such as antigen uptake, processing, and escape to the cytosol.

### A1 triggers intra-endosomal ROS production through NOX activation

**Figure 3:**
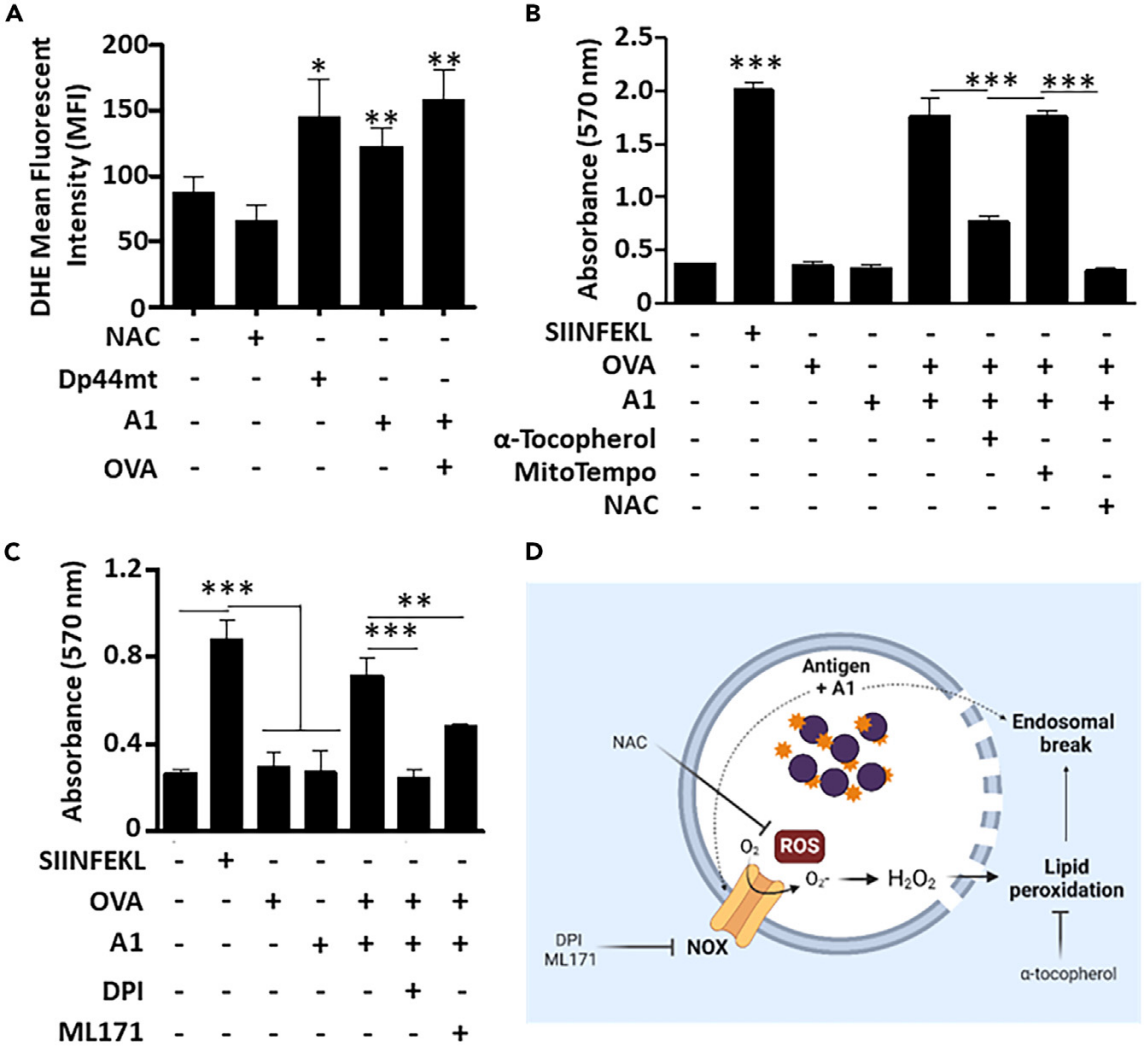
The A1-induced antigen cross-presentation capacity increases and requires ROS production (A) Flow cytometry assessment of ROS production by MSCs in response to A1 using the dye DHE. NAC was used as the negative control whereas Dp44mt was used as a positive control. (B) Antigen cross-presentation assay performed to investigate the effect of neutralizing ROS on A1-related activity. (C) Same as (B) except that it was conducted using NOX inhibitors. (D) A graphical abstract summarizing the mechanistic insights of A1-driven endosomal disruption obtained from the data above. Results are presented as average mean with standard deviation (S.D.) error bars, and statistical significance is represented with asterisks: ∗*p* ˂ 0.05, ∗∗*p* ˂ 0.01, ∗∗∗*p* ˂ 0.001.

To explore other commonly observed events in DCs capable of cross-presenting, we next assessed ROS production using dihydroergotamine (DHE), a cell-permeable superoxide indicator dye. Interestingly, A1 triggers similar ROS production levels compared to Dp44mt (Figure 3A), a ROS-inducing agent used as a positive control. To assess the importance of this observation, we next evaluated the neutralizing impact of different antioxidants on antigen cross-presentation. Treatment with N-acetylcysteine (NAC), a general cysteine donor scavenging free radicals, completely abolished cross-presentation (Figure 3B). In contrast, treatment with MitoTEMPO, a mitochondria-targeted antioxidant, did not affect B3Z activation, therefore excluding a role for mitochondria-driven ROS (Figure 3B). Interestingly, however, co-incubation with α-tocopherol, a vitamin-E derivative capable of blocking lipid peroxidation, significantly decreased MSCs ability to cross-present (Figure 3B). As a role for mitochondrial-derived ROS is inapparent, we next focused on the possible activation of NOX, which can be found within endosomes as reported in other specialized DCs subsets.^25^^,^^26^^,^^27^ To do so, A1-pulsed MSCS were co-treated with generic (DPI) versus NOX1-specific (ML171) inhibitors. Although ML171 partially decreased B3Z activation, the use of DPI completely suppressed antigen cross-presentation (Figure 3C). These findings indicate that A1 leads to intra-endosomal ROS production via NOX, which causes endosomal break via lipid peroxidation (Figure 3D).

### A1 induces protein aggregation and drives the unfolded protein response (UPR)

**Figure 4:**
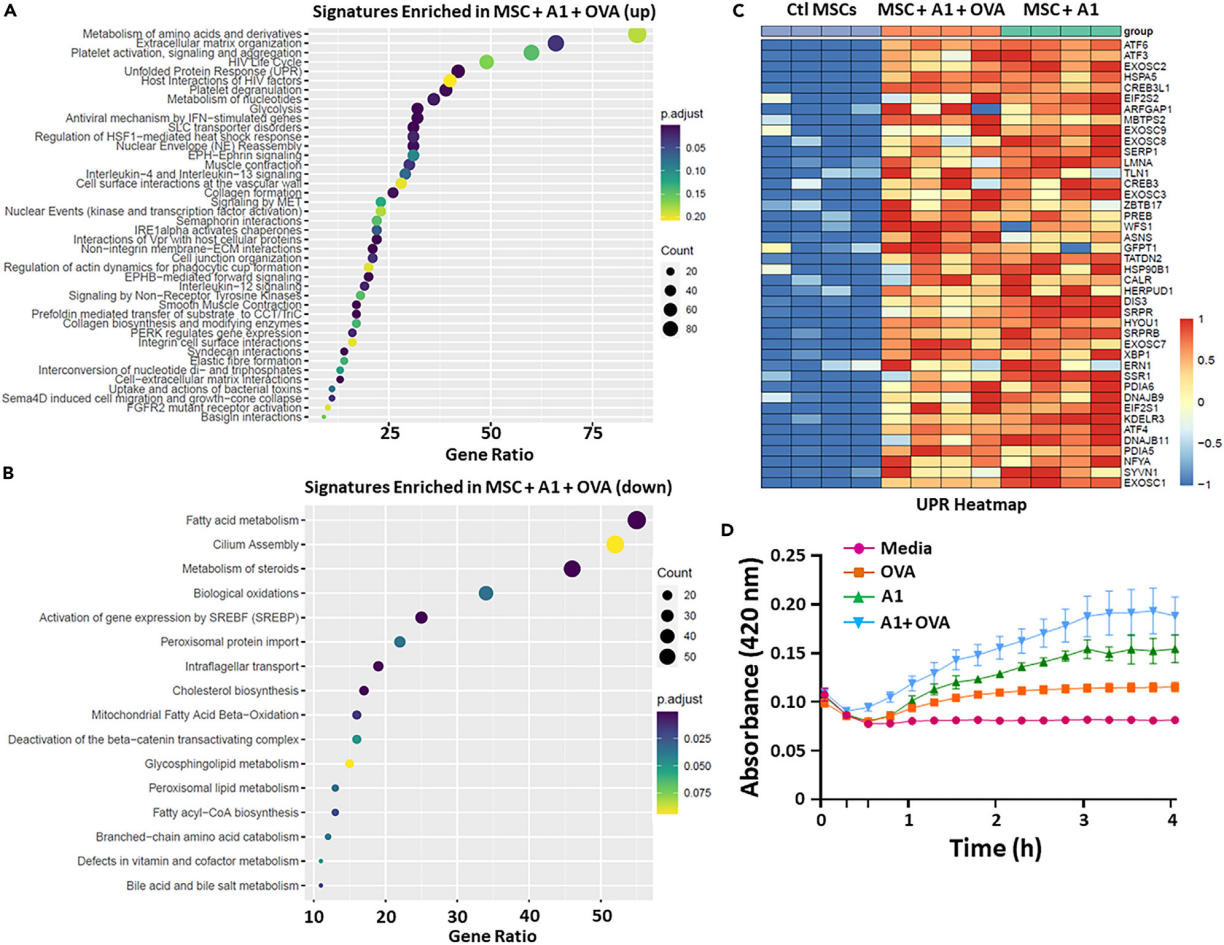
Molecular characterization of the ARMs List of top Reactome pathways that are enriched for both up-regulated (A) and down-regulated (B) genes in the A1 treated group versus control MSCs. The circle’s color corresponds to adjusted p values; the size of the circles corresponds to the ratio count of genes in the tested set. (C) A representative unfolded-protein response (UPR) heatmap displaying the genes that contribute the most to the pathway enrichment and modulated in response to A1 treatment (FDR <5%); gene expression is scaled between −1 and +1, followed by color code indicated in the figure. (D) A turbidity assay to evaluate the A1 capacity to form protein aggregation when mixed with the OVA protein. The groups and controls are depicted according to the color code. Results are presented as average mean with standard deviation (S.D.) error bars, and statistical significance is represented with asterisks: ∗*p* ˂ 0.05, ∗∗*p* ˂ 0.01, ∗∗∗*p* ˂ 0.001. See also Figures S4–S6.

To better understand the impact of A1 on MSCs, we conducted a whole transcriptome analysis by comparing control to A1- or A1/OVA-treated MSCs. Besides the strong correlation between the two A1 conditions (Figure S4A), indicating a similar expression profile, A1 treatment leads to more than 1,500 differentially expressed genes (DEGs) (Figure S4B). Among the upregulated processes in A1-treated MSCs cells, we observe UPR, metabolism of nucleotides, glycolysis, and regulation of HSF-1-mediated heat shock response (Figure 4A) whereas pathways related to fatty acid or steroid metabolism, cholesterol biosynthesis as well as bile acid and bile salt metabolism were downregulated (Figure 4B). Since misfolded, damaged, or aggregated proteins can all lead to endoplasmic reticulum (ER) stress and UPR upregulation, including the expression of sensory pathways related to the ER stress such as ATF4, ATF6, and XBP1 (Figure 4C), a turbidity assay was conducted using OVA admixed with A1. As shown in Figure 4D, A1 on its own aggregates, an observation that was further enhanced when admixed with the OVA protein. Besides UPR-related changes (Figures 4C, S4C, and S5), molecular profiling of A1-treated cells revealed activation of interferon (IFN)-stimulated genes and interleukin (IL)-12 signaling (Figures 4A and S6A). Consistent with these observations, secretome analysis using Luminex revealed increased secretion of pro-inflammatory mediators such as G-CSF, GM-CSF, IL-6, TNF-alpha, and IL-12 (Figure S6B). Based on the extensive impact of A1 on the molecular and physiological profile of MSCs, we elected to designate these cells as A1-reprogrammed-MSCs (ARMs).

### Vaccination using ARM cells induce potent antitumoral responses

**Figure 5:**
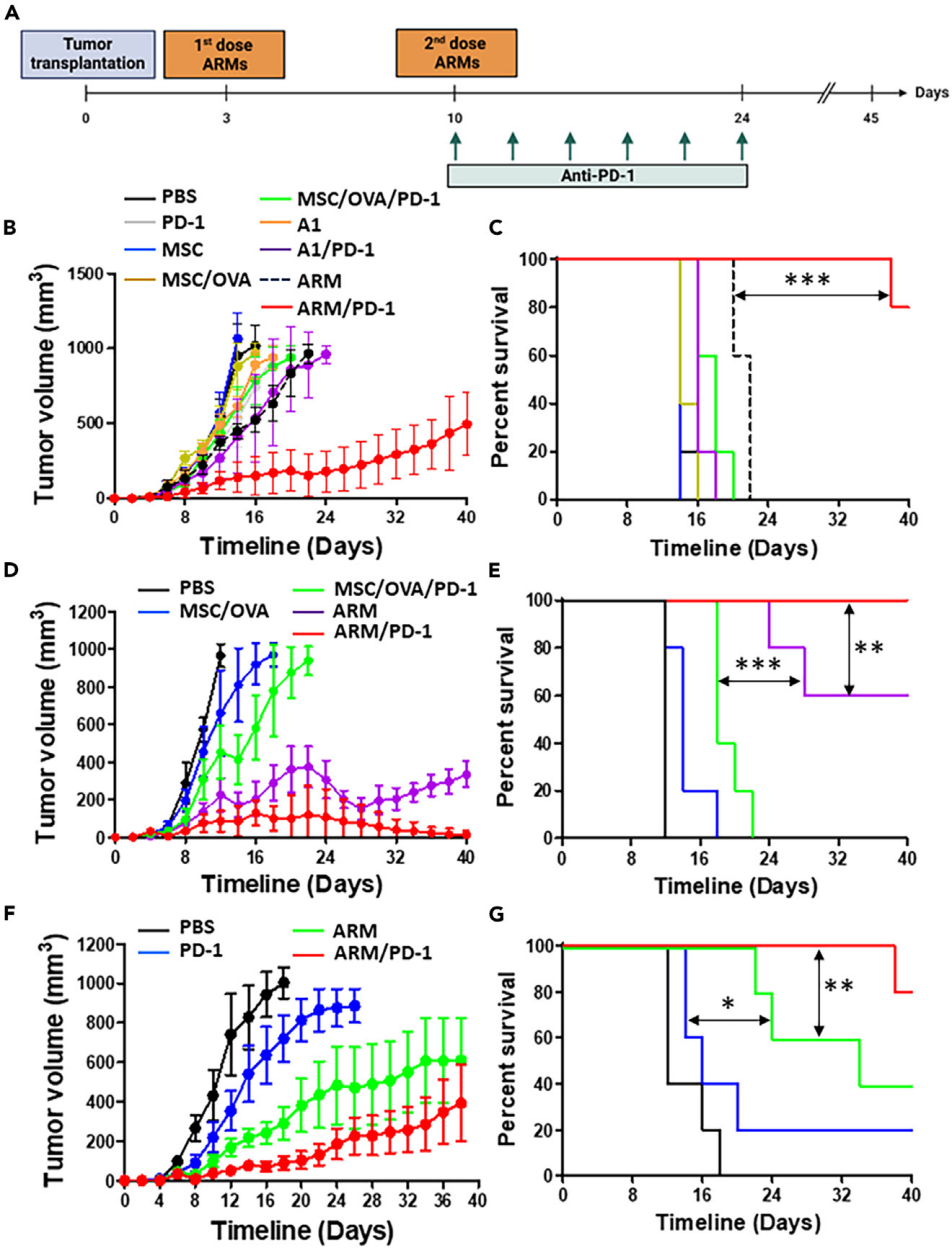
Therapeutic vaccination using the ARM vaccine can induce regression of established tumors (A) Experimental design represented by the timeline of the ARM therapeutic vaccination as a monotherapy or combination treatment approach with the ICI anti-PD-1. (B) Evaluation of E.G7 tumor growth in response to syngeneic ARM vaccination (MSCs were obtained from and administered to C57BL/6 mice). The group conditions are indicated by the color code. E.G7 is an OVA-expressing T cell lymphoma, and the protein OVA is used as the stimulating antigen. (C) Kaplan-Meier survival curve of the experiment shown in (B). (D) Evaluation of E.G7 tumor growth in response to allogeneic ARM vaccination (MSCs were obtained from BALB/c mice and administered to tumor-bearing C57BL/6 mice), using OVA as the stimulating antigen. (E) Kaplan-Meier survival curve of the experiment shown in panel D. (F) Evaluation of B16 tumor growth in response to allogeneic ARM vaccination using B16 lysate as stimulating antigen. B16 is a melanoma cell line, and the protein lysate of B16 cells was used as the pulsing antigen. (G) Kaplan-Meier survival curve of the experiment shown in (F). For panels (B–E), n = 5/group. PD-1 refers to the antibody anti-PD-1. Results are presented as average mean with standard deviation (S.D.) error bars, and statistical significance is represented with asterisks: ∗*p* ˂ 0.05, ∗∗*p* ˂ 0.01, ∗∗∗*p* ˂ 0.001. See also Figure S7.

To evaluate the antitumoral capacity of the ARM cells, we conducted a series of vaccination studies against solid tumors. As illustrated in the experimental design (Figure 5A), SC delivery of syngeneic ARM cells was performed 3 days post-EG.7 tumor implantation followed by a second dose a week later as a monotherapy or in combination with anti-PD-1. In contrast to all tested conditions, co-administrating the ARM vaccine with anti-PD-1 elicits a prominent tumor control response (Figure 5B), with almost all mice (80%) surviving by day 40 (Figure 5C). When repeated under allogeneic settings, however, ARM administration as a monotherapy triggered a superior response (Figures 5D and 5E) compared to the same treatment group under syngeneic settings. Nevertheless, combining the allogeneic ARM cells with anti-PD-1 cured all treated animals (Figure 5D) with complete survival obtained by day 40 (Figure 5E). No differences in antitumoral responses were observed when allogeneic ARM cells were delivered using the SC versus IT route (Figures S7A and S7B). On the other hand, the ARM effector response was dose-dependent, with a loss in therapeutic potency observed using the 1 x 10^4^ cell dose (red line) in contrast to using 5 x 10^5^ cells (green line—Figures S7C and S7D).

All proof-of-concept studies conducted so far used OVA as a single pre-defined antigen. This approach may not be clinically viable for two main reasons. First, there is currently no known tumor-specific antigen (TSA) shared by a large portion of the population and capable of inducing potent antitumoral responses.^34^ Second, past studies showed that targeting a single antigen is most likely prone to elicit tumor editing/escape over time.^2^ Since we have a cell type capable of effectively cross-presenting soluble antigens, we elected to overcome these limitations using tumor lysate preparations. This approach not only allows the presentation of multiple unknown neoantigens or TSAs, but it also permits the generation of an immune response specific to each patient/cancer indication. When tested against the B16 melanoma model, for instance, the immune response generated following allogeneic ARM cell administration (monotherapy) effectively controlled tumor growth (Figure 5F) with 40% survival rate obtained over 40 days (Figure 5G). Co-administration with anti-PD-1, on the other hand, significantly improved the antitumoral response (Figure 5F), doubling therefore the survival rate (Figure 5G). Overall, our findings convey two important messages. First, the ARM vaccine can indeed induce potent antitumoral responses even when pulsed with tumor lysate. Second, the developed approach is versatile and can be adapted to different cancer types if given access to tumor tissues/biopsies.

### Dimerization improves the molecular stability of A1 while eliciting similar antitumoral effects

**Figure 6:**
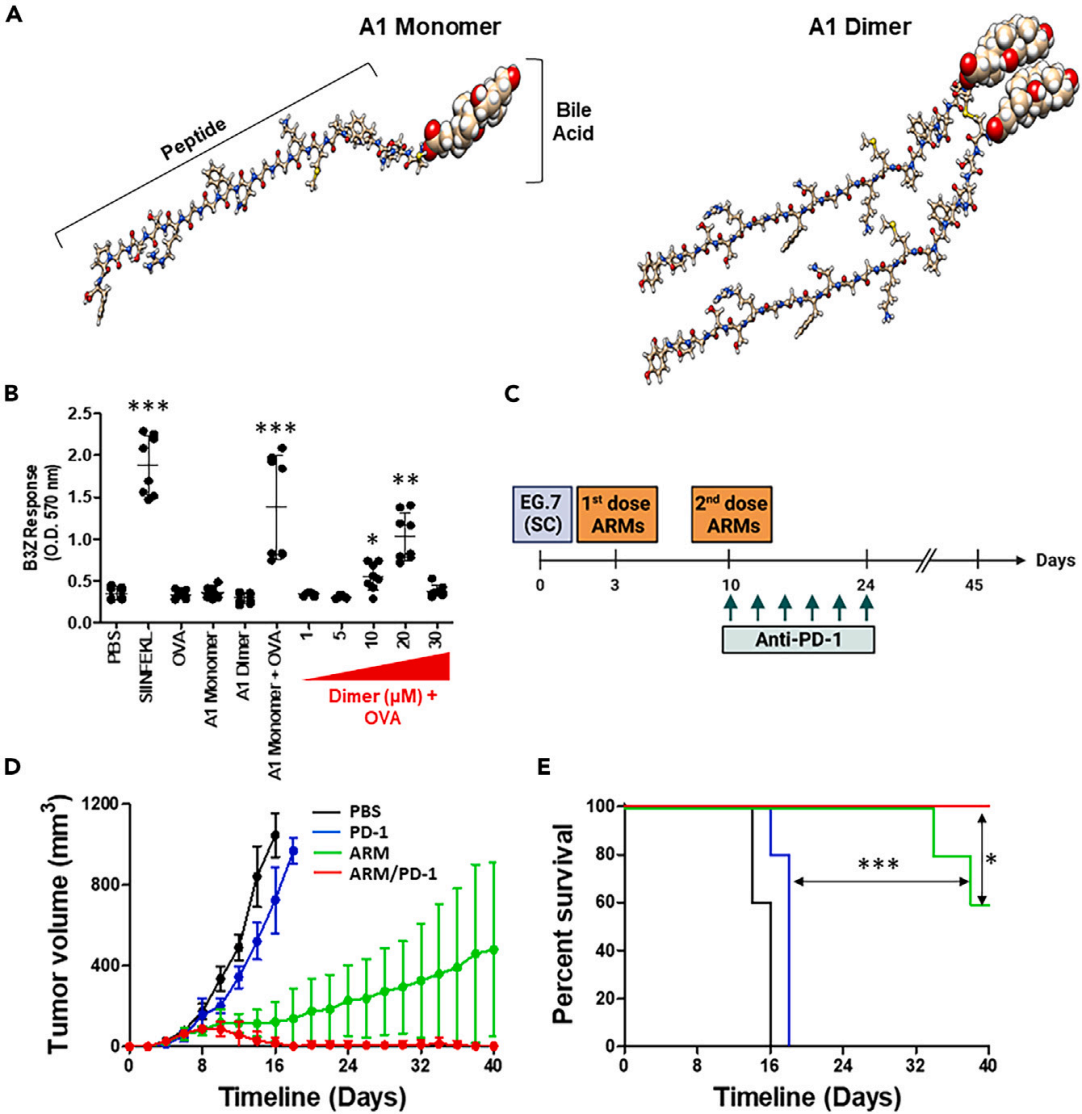
Optimizing the therapeutic potency of the ARM vaccine using a dimeric form of A1 (A) Structure of the A1 monomer (left) versus dimer (right). (B) Comparing the antigen cross-presentation capacities of ARM cells generated in response to the monomer versus different concentrations of dimer form of A1. (C) Experimental design used for the therapeutic vaccination used to test the ARM cells generated using the A1 dimer, as a monotherapy or combination treatment approach with the ICI anti-PD-1. (D) EG.7, an OVA-expressing T cell lymphoma, tumor growth in response to allogeneic ARM vaccination. The vaccination consisted of BALB/c MSCs pulsed with A1 dimer and the antigen OVA, administered to E.G7-bearing C57BL/6 mice. (E) Kaplan-Meier survival curve of the experiment shown in (G). For (B), n = 6/group. For (D and E), n = 10/group. Results are presented as average mean with standard deviation (S.D.) error bars, and statistical significance is represented with asterisks: ∗*p* ˂ 0.05, ∗∗*p* ˂ 0.01, ∗∗∗*p* ˂ 0.001.

Throughout our studies, we noticed a decrease in A1 stability over time, most likely due to the oxidation of exposed amino acid residues. In addition, the manufacturing of the monomeric A1 form can result in the generation of non-specific “contaminants” that can impair its overall activity. To bypass these limitations, we engineered a dimer of A1, where two molecules are linked together through the NLS peptide (Figure 6A). The dimer remained active, and its ability to induce cross-presentation by MSCs was observed at a much lower dose (20 μM in contrast to 50 μM with the monomeric form) with a decrease in ARM viability at higher concentrations (Figure 6B). When tested in the context of therapeutic vaccination against the E.G7 lymphoma model, the response induced by the ARM vaccine was improved (60% versus 40% survival with the A1 monomer—Figure 5E) as a monotherapy and was further enhanced when combined with anti-PD-1 (Figures 6D and 6E). These results demonstrate that the A1 dimer is indeed active, requiring lower pulsing concentrations (20 μM compared to 50 μM) while improving the therapeutic potency of the allogeneic ARM vaccine.

## Discussion

While DC-based vaccination showed moderate therapeutic effects against cancer, their clinical use is hindered by their limited availability and associated manufacturing hurdles.^1^^,^^35^ Our study proposes an alternative vaccination platform utilizing the ARM cells, which not only synergize with immune checkpoint inhibitors leading to tumor regression and increased survival rates, but also offers a compelling and versatile strategy adaptable to the generation of a broader scope of cancer vaccines. More specifically, our strategy consists of pharmacological reprogramming MSCs to cross-present antigens. By admixing A1 with a defined or a set of mixed antigens (e.g., tumor lysate), protein aggregation takes place and is captured in endosomes by MSCs. At that stage, A1 stimulates NOX to produce ROS resulting in lipid peroxidation and cargo release in the cytosol. Since protein aggregation is seen as a “danger signal,” the cell responds through UPR upregulation, which ends up targeting captured proteins for proteasomal degradation. Generated peptides are then presented on the cell surface to responding CD8 T cells while the pro-inflammatory cytokine profile of ARM cells supports the ongoing immune response.

Besides enhanced antigen uptake and processing, one of the most salient observations made in this study relates to ROS production. Surprisingly, the origin of ROS was found to be unrelated to the mitochondria but instead involves NOX activation, a process previously described in cross-presenting cDC1.^25^^,^^26^^,^^27^ Intra-endosomal production of ROS in this context has two crucial advantages. First, it prevents endosomal acidification, which would impair protease activation, avoiding, therefore, non-specific degradation of the captured cargo.^25^^,^^26^^,^^27^ Second, ROS production triggers lipid peroxidation, which destabilizes and ruptures endosomal membranes, thereby releasing captured intact antigens into the cytosol.^24^ Intriguingly, the parent Accum molecule could not promote endosome-to-cytosol escape (Figure 2G) nor antigen cross-presentation in MSCs when admixed with the OVA antigen. This indicates a distinct gain of function for A1 related to ROS-induced endosomal disruption. Although our model provides direct links between A1, NOX activity, and ROS production, additional investigations are needed to confirm whether lipid peroxidation acts synergistically with an additional A1-induced membrane perturbation mechanism or if it is the sole inducer of endosomal disruption.

Transcriptomic analysis revealed that A1 triggers the upregulation of both the heat shock factor 1 (HSF1) and UPR, two key players in cellular stress response and the re-establishment of protein homeostasis. Proteostasis is a vital cellular process and is tightly regulated to avoid problems that could lead to cellular dysfunction and/or death.^36^ The conducted turbidity assay, combined with UPR, corroborates the hypothesis that A1 induces protein aggregation.^36^ In consequence, UPR activation has two possible outcomes: restoring homeostasis or inducing apoptosis.^37^ Interestingly, the ubiquitin-proteasome system is tightly related to ER stress.^38^ Hence, stimulation of the proteasomal machinery could lead to the activation of antigen presentation machinery,^39^ contributing to the efficiency of ARM cells in cross-presenting antigens. In cross-presenting CD8α^+^ DCs, constitutive activation of ER genes was found to be essential for maintaining proper protein homeostasis, particularly during increased demands for cytokine and MHC production,^40^^,^^41^ which correlates to the efficiency in their role as professional APCs. Although we did not observe an increase in MHC expression, our secretome analysis unveiled an increase in the secretion of various cytokines by ARM cells some of which exhibiting pro-inflammatory functions. This could suggest a connection between ER signaling and the antigen-presenting capabilities of the ARM cells, resembling professional APCs.

The crosstalk between ER stress, UPR, and lipid metabolism has been debated in the literature.^42^^,^^43^ Beyond the classical view that the UPR induces upregulation of lipogenesis to support ER membrane extension, recent studies demonstrated how the UPR could also have implications in lipid and sterol synthesis, promoting lipolysis by activation of intermediary sensors in the UPR pathway that alter lipid enzymes.^43^^,^^44^ Moreover, it is possible that A1-induced lipid peroxidation leads to cytosolic lipid accumulation, causing a state of lipid overload and downregulation of fatty acid metabolism. The structure of the A1 molecule, containing cholic acid, may also play a role in reducing cholesterol biosynthesis as a response to lipid overload since cholesterol is a precursor of cholic acid, potentially resulting in the impact observed in bile acid metabolism. As a result, various cellular events may have induced lipid-related pathways to reduce lipid accumulation and restore cellular homeostasis.

The MSC mode of action remains a highly debated topic within the field of cell therapy.^13^ Initially, the therapeutic function of MSCs as immunosuppressants was attributed to their secretome.^13^^,^^45^ However, elegant studies have shown that MSCs undergo apoptosis after administration, either in a nonspecific manner through bystander effect of activated cytotoxic cells^46^ or by events unrelated to cell function, such as nutrient/growth factor deprivation and mechanical stressors.^47^ Then, apoptotic MSCs attract phagocytes, which capture their particles for clearance through efferocytosis. This process was demonstrated to be required for MSC’s therapeutic effect.^46^^,^^47^ In the ARM vaccination scenario, it is possible to stipulate that allogeneic ARMs undergo apoptosis and phagocytes capture them, process them, and present their peptides, subsequently interacting with host-derived responding T cells. The role of MSC efferocytosis is further supported by a study from our group showing how IRMs (genetically modified MSCs expressing the immunoproteasome complex) could have their antitumoral therapeutic effect hindered by the depletion of phagocytes.^48^ It is also possible that the cellular stress induced by the A1 molecule turns the ARMs more prone to apoptosis after their administration to the host, which could contribute to their therapeutic benefit.^47^ Although the ARM cells may not directly activate cytotoxic cells, their ability to cross-present antigens make them effective vehicles for transporting processed tumor antigens, initiating a cascade of cell activation, and eliciting a robust immune response against solid tumors. Nevertheless, the superior response observed in allogeneic versus syngeneic vaccination settings may be attributed to the more efficient mobilization of phagocytes due to allorecognition. The latter hypothesis is supported by the fact that allogeneic vaccination increases the immunogenicity of cancer vaccines.^49^^,^^50^ Furthermore, the use of allogeneic cell-based vaccines offers manufacturing advantages, positioning the ARM cells as a potential versatile off-the-shelf vaccine.

Despite the yet unclear mechanism of action of the ARM vaccine and its role as an antigen carrier, it is evident that combinatorial treatment with anti-PD-1 enhances the immune response against transplanted tumors in mice. T cell activation is an intricately regulated process involving peptide-MHC engagement of the T cell receptor and other stimuli. However, upon activation, coinhibitory checkpoints, including PD-1, can be induced to regulate T cells, hindering their antitumoral activity during encounters with PD-L1-expressing cancer cells.^51^ While PD1 pathway inhibitors have revolutionized cancer treatment, incomplete responses in the majority of patients^52^ suggest that combinatorial treatments may enhance or restore immunotherapy potential.^51^ The observed enhanced antitumoral response in the combination therapy group (ARM vaccine and anti-PD-1) implies a synergistic effect. This could be attributed to the engagement of a new repertoire of T cells induced by the ARM vaccine, coupled with the prevention of T cell inhibition by targeting the PD-1 inhibitory pathway. This encourages further assays to be performed to investigate ARM’s effects *in vivo* and CD8 T cell activation, along with assessing the contribution of checkpoint blockade, thereby characterizing the interplay among the therapies.

In sum, utilizing cells from unrelated donors allows the establishment of a predefined “master” and/or “working” cell banks, readily available for patient use is an appealing cell therapy modality.^53^^,^^54^ This eliminates the need for obtaining and expanding MSCs for each patient, which would require specialized infrastructure and skilled personnel at every treatment center, thereby streamlining the treatment process and decreasing the number of invasive procedures.^53^ The personalized and simplified off-the-shelf approach proposed in this study holds promise for tailoring treatments to individual patients and tumor types while offering manufacturing advantages. It further lays the groundwork for further optimization and demands future investigations to unravel the precise mechanism of action, paving the way for potential advancements and applications in the future.

### Limitations of the study

While this study provides a promising candidate for cancer vaccine development, there are certain limitations and high potential for further exploration. For example, the used *in vitro* antigen presentation assays do not provide a definitive confirmation that T cell activation will take place *in vivo.*^29^ Similar *in vitro* antigen cross-presentation assay models also do not confirm that the analyzed cells are indeed cross-presenting protein directly to T cells *in vivo*. To address these concerns, future studies would include analysis of the T cell population post-vaccination and immunopeptidome studies, respectively. Additional studies investigating the impact of administrating clodronate to induce phagocyte depletion as a means to assess its impact on efferocytosis contribution to vaccination outcomes are needed.^48^ Furthermore, the study reveals the role of A1 in inducing endosomal break, however, a deeper understanding of the mechanistics related to this capacity requires further investigation. Moreover, this vaccination strategy should be assessed in different cancer models, focusing on solid tumors as they facilitate antigen acquisition through cell lysates from tumor surgical resections for stimulation, and on human MSCs, to pave the road for future clinical applications.

## STAR★Methods

### Key resources table

**Table:** 

REAGENT or RESOURCE	SOURCE	IDENTIFIER
**Antibodies**		

Alexa Fluor® 647 Mouse Anti-Mouse H-2K[b]	BD Biosciences	Cat# 562832; RRID: AB_2737824
Alexa Fluor® 647 Rat Anti-Mouse I-A/I-E	BD Biosciences	Cat# 562367; RRID: AB_11152078
RecombiMAb anti-mouse PD-1 (CD279)	BioXCell	Cat# CP157; Clone: RMP1-14-CP157
CD44	BD Biosciences	Cat# 561862; RRID: AB_398661
CD45	BD Biosciences	Cat# 553081; RRID: AB_394611
CD73	BD Biosciences	Cat# 567215; Clone TY/11.8
CD90	BD Biosciences	Cat# 561409; RRID: AB_10683163

**Chemicals, peptides, and recombinant proteins**		

Accutase®	Sigma-Aldrich	Cat# A6964
Albumin from chicken egg white	Sigma-Aldrich	Cat# A5503-50G
Annexin V Binding Buffer	BioLegend	Cat# 422201
APC Annexin V	BioLegend	Cat# 640920
Bradford Protein Assay Dye Reagent Concentrate	Bio-Rad	Cat# 5000006
Chlorophenol red-β-D-galactopyranoside (CPRG)	Sigma-Aldrich	Cat# 10884308001
Cytochrome c from equine heart	Sigma-Aldrich	Cat# C2506
Dihydroethidium (DHE)	Invitrogen	Cat# D11347
Diphenyleneiodonium Chloride (DPI)	Sigma-Aldrich	Cat# 300260
DL-alpha-Tocopherol	ThermoFisher	Cat# A17039-18
Dp44mT	Sigma-Aldrich	Cat# SML0186
DQ™ Ovalbumin	Invitrogen	Cat# D12053
MitoTEMPO	Sigma-Aldrich	Cat# SML0737
ML171	Millipore Corp	Cat# CAS 6631-94-3
N-Acetyl-L-cysteine	Sigma-Aldrich	Cat# A7250
Ovalbumin, Alexa Fluor™ 647 Conjugate	Invitrogen	Cat# O34784
SIINFEKL peptide	GenScript	Cat# RP10611

**Critical commercial assays**		

RNeasy Mini Kit	QIAGEN	Cat# 74104
EasySep Mouse CD8a Positive Selection Kit	StemCell Technologies	Cat #: 18753
EasySep Mouse CD8a Positive Selection Kit II	StemCell Technologies	Cat#:18953
Mouse IFN-gamma Quantikine ELISA Kit	R&D systems	Cat#: MIF00

**Deposited data**		

Wild-type MSCs versus ARM cells RNA-seq data	Gene Expression Omnibus	Accession "GSE247278"

**Experimental models: Cell lines**		

Mouse: E.G7-OVA [derivative of EL4]	ATCC	Cat# CRL-2113; RRID: CVCL3505
Mouse: B16-F0	ATCC	Cat# CRL-6322; RRID: CVCL_0604
Mouse: B3Z	Gift from Dr. Etienne Gagnon	N/A

**Experimental models: Organisms/strains**		

Mouse: BALB/cAnCrl	Charles River	Strain code: 028
Mouse: C57BL/6NCrl	Charles River	Strain code: 027
Mouse: OT1 (C57BL/6-Tg(TcraTcrb)1100Mjb/J)	The Jackson Laboratory	Strain code: 003831

**Software and algorithms**		

FlowJo™ v10	FlowJo	https://www.flowjo.com/solutions/flowjo/downloads
GraphPad Prism	Dotmatics	https://www.graphpad.com/
BioRender	BioRender	https://www.biorender.com/
Bioinformatics software(s)	R statistical programming, Other packages: Ggplot2 ClusterProfiler	https://cran.r-project.org/web/packages/ggplot2/index.html https://bioconductor.org/packages/release/bioc/html/clusterProfiler.html

**Other**		

Amicon® Ultra-15 Centrifugal Filter Unit	Millipore	Cat# UFC910024

### Resource availability

#### Lead contact

Further information and requests for resources and reagents should be directed to and will be fulfilled by the Lead Contact, Moutih Rafei (moutih.rafei.1@umontreal.ca).

#### Materials availability

Some of the reagents used in this study will be made available on request, but we may require a payment and/or a completed Materials Transfer Agreement if there is potential for commercial application.

#### Data and code availability

• RNA-seq data have been deposited at GEO and are publicly available as of the date of publication. Accession numbers are listed in the key resources table.
• Any additional information required to reanalyze the data reported in this paper is available from the lead contact upon request.
• This paper does not report an original code. Any analyses applied are based on previously available software and established R packages, primarily, custom R scripts (https://www.R-project.org/), ggplot2 and clusterprofiler (PMID: 22455463).

### Experimental model and study participant details

#### Mice strains

For all experiments, six- to ten-week-old female C57BL/6 and BALB/c were purchased from Charles River (Senneville, QC, Canada). Six- to ten-week-old female OT-I mice were purchased from Jackson Laboratories (Bar Harbor, ME, USA). The mice were housed and maintained in accordance with the guidelines approved by the Animal Care Committee of Université de Montréal in a pathogen-free environment at the animal facility of the Institute for Research in Immunology and Cancer (IRIC). Animal protocols were approved by the Animal Care Committee of Université de Montréal (protocol # 22-065).

#### Cell lines

B16F0 and E.G7 cells were purchased from ATCC. B16F0 cells were maintained in Dulbecco’s modified Eagle’s medium (DMEM) supplemented with 10% FBS. E.G7 and B3Z cells were cultured in RPMI 1460 supplemented with 10% FBS, 50 U/mL Penicillin-Streptomycin, 2 mM L-glutamine, 10mM HEPES, 1mM Sodium Pyruvate, and 0.5 mM β-Mercaptoethanol. E.G7 were kept under selection using 0.4 mg/ml of G418. All cells were maintained at 37°C in a 5% CO2 incubator. All cell culture media and reagents were purchased from Wisent Bioproducts (St-Bruno, QC, Canada).

#### Generation of bone-marrow-derived MCSs

Wild type bone marrow derived MSCs were collected as previously detailed.^12^ To obtain MSCs, the bone marrow was obtained from the femurs of 6-8 weeks old female C57BL/6 or BALB/c mice by flushing with Alpha Modification of Eagle’s Medium (AMEM) supplemented with 10% FBS, and 50 U/mL Penicillin-Streptomycin in 10 cm^2^ cell culture dish. After 48 hours, non-adherent cells were removed by changing the media after 24 hours followed by gently changing the median every 3 to 4 days.^55^ When the cells reached 80% confluency, adherent cells were collected using 0.25% Trypsin and expanded until forming a homogenous population before their assessment for the expression of surface markers CD44, CD45, CD73, and CD90, using flow cytometry. The confirmed MSCs were expanded and stored in liquid nitrogen at passage number 9 or 10. The pleiotropic differentiation capacity of generated MSCs was evaluated by inducing their osteogenic and adipogenic differentiation as reported previously.^55^ Osteogenic differentiation of MSCs was induced by plating them at 60% confluency, once adherent, the cells were cultured for 3-4 weeks in AMEM media supplemented with 10% FBS in addition to β-glycerol phosphate (10 mM), dexamethasone (10-8 M), and ascorbic acid 2-phosphate (5 μg/mL), changing the media every 2-3 days.^56^ Thereafter, osteogenic differentiation was validated by staining calcium deposits using Alizarin Red S. by washing the cells using with phosphate-buffered saline (PBS), followed by incubation for 5 minutes in 2% Alizarin Red S solution (pH adjusted to 4.1 using ammonium hydroxide), then rinsed with distilled H_2_O.^55^ For adipogenic differentiation, the MSCs were plated at 50% confluency then kept in AMEM supplemented with 10% FBS, indomethacin (46 μM), 3-isobutyl-methylxanthine (0.5 mM), dexamethasone (1 μM), and insulin (10 μg/mL), changing the media twice during 7 days.^57^ At the end of the differentiation period, oil droplets within differentiated adipocytes were visualized using Oil Red O. The cells were first fixed for 1 hour, at room temperature with 4% paraformaldehyde, followed by staining for 10 minutes using Oil Red O solution. The solution was prepared by mixing Oil Red O (dissolved at 3.75% in isopropanol) and 2 parts distilled H_2_O. At the end of incubation time, the cells were rinsed with distilled H_2_O.^55^^,^^56^ The cells were visualized via transmitted light and imaged using EVOS® FL cell imaging microscope (Thermo Fisher Scientific).

#### Vaccination studies

To generate the cellular vaccine, the MSCs were pulsed with fresh media containing 0.5-1 mg/ml of stimulating antigen with or without A1 (A1 monomer: 50 μM; A1 dimer: 20 μM) for 6 hours. At the end of pulsation time, the cells were washed with PBS, detached using Accutase®, then counted and washed three times with PBS, then resuspended to obtain 5 x 10^5^ cells in 100μl. Tumor cells were similarly counted and washed three times using PBS. The cells were kept on ice prior to injection. To evaluate the therapeutic properties of the vaccine under syngeneic regimen, female C57BL/6 mice (n=5-10/group) were subcutaneously (SC) injected with 5 x 10^5^ E.G7-OVA cells at day 0 on the hind. At days 3 and 10, the mice were intratumorally (IT) or SC-injected (near the tumor) with 5 x 10^5^ MSCs pulsed with 1 mg/ml of OVA and 50 μM A1 monomer. Control animals received 5 x 10^5^ tumor cells alone. To assess the effectiveness of the therapeutic vaccine as a combination therapy with immune-checkpoint inhibitor anti-PD-1, starting day 10, the mice start receiving intraperitoneal (IP) injections of the antibody or its isotype at 200 μg/per dose every 2 days for a total of 6 doses over two weeks. For allogeneic vaccination, three groups tested had C57BL/6 mice transplanted with E.G7-OVA followed by vaccination using BALB/c-derived MSCs, pulsed for 6 hours either with: i) 1 mg/ml of OVA and 50 μM A1 monomer, ii) 0.5 mg/ml of B16-lysate and 50 μM A1 monomer, or iii) 1 mg/ml of OVA and 20 μM A1 dimer. All animals were followed for tumor growth using a digital caliper for 6 weeks or until reaching endpoints (ulceration or a tumor volume ≥ 1000 mm^3^).

### Method details

#### Phenotypic analysis by flow cytometry

For phenotypic confirmation of cell surface markers, the cells were collected, counted, and washed with PBS twice. To stain surface markers, the cells were resuspended at the density of 10^5^ cells/ml in cold 2% FBS in PBS and incubated with flow cytometry antibodies or their isotypes diluted according to manufacturer’s instructions for 30 min at 4°C in the dark. To remove excess antibodies, the stained cells were washed twice with cold 2% FBS in PBS buffer. Finally, the cells were resuspended in 400μl of cold 2% FBS in PBS and kept on ice in the dark until they were acquired by BD FACS Diva on CANTOII. The obtained data was analyzed using FlowJoV10.

#### Antigen cross-presentation assay

To screen the different Accum™ analogues for effect on MSCs ability to act as APC, a cross-presentation assay was used where 25 x 10^3^ MSCs were seeded per well in a 24-well plate. On the following day, the cells were pulsed by adding fresh media containing 1 mg/ml of OVA in addition to different Accum® variants (at 50 μM). At the end of the pulsing period, the cells were washed with PBS, then 5 x 10^5^ B3Z cells were added per well. The co-culture was incubated for 17-19 hours. The media was then removed, and the cells washed once with PBS and lysed using lysis buffer (tris base, CDTA, glycerol and triton X-100) and shaken for 20 minutes at room temperature. Cell lysate is then incubated with a CPRG solution (containing CPRG, disodium phosphate, monosodium phosphate, potassium chloride, magnesium sulfate) protected from light- for 24 hours at 37°C.^33^ The optical density signal was detected at wavelength 570 nm using a SynergyH1 microplate reader (Biotek, Winooski, VT, United States). For the assay using OT-T-derived CD8 T cell, the cells were seeded at 25 x 10^3^ cells per well in a 24-well plate. On the following day, the cells were washed and pulsed with the antigen of interest (1 mg/ml of OVA or 1 μg/ml of the SIINFEKL peptide). At the end of the pulsing period, the cells were washed to remove excess antigen and co-cultured with 10^6^/ml CD8 T-cells purified from the spleen of OT-I male mice (6-10 weeks old) using the CD8α^+^ positive isolation kit according to the manufacturer’s protocol. After 72 hours, supernatants were collected, and centrifuged for 5 min at 1500 rpm, 4°C. Clear supernatants were used to quantify IFNγ levels by ELISA (R&D).

To evaluate the effects of ROS neutralization on A1-induced cross-presentation, the same antigen cross-presentation assay described above was performed with the selected inhibitors added at the same time as the A1 molecule. After 6 hours of incubation, the cells were washed and 5 x 10^5^ B3Z cells were added per well. In addition to using NAC (5 mM) as a general ROS inhibitor, MitoTEMPO (10 μM) was used as a specific mitochondrial ROS inhibitor whereas α-tocopherol (2 mM) was tested as a blocker for lipid peroxidation.

#### Monitoring antigen uptake and processing

To evaluate OVA uptake, 5 x 10^4^ MSCs were seeded per well in a 12-well plate. On the following day, the cells were first treated with 1 μg/ml of Alexa Fluor® 647-conjugated OVA admixed with the Accum® variant A1 for 3 hours at 37°C. The cells were then detached, collected, and washed with cold PBS containing 2% FBS and assessed for their fluorescence intensity by flow cytometry. To evaluate antigen processing, MSCs were incubated with 10 μg/mL DQ® ovalbumin admixed with A1 at 37°C. One hour later, cells were washed, and regular media was added for 3 hours. At the end of the indicated incubation, cells were collected and washed with cold PBS containing 2% FBS. Fluorescence was monitored using BD FACS Diva on CANTO II.

#### Assessing endosomal escape

Endosomal leakage was assessed using an apoptosis assay described previously.^24^ Briefly, 10^5^ MSCs were seeded per well in a 6-well plate and supplemented with 10 mg/ml of exogenous Cyt-C for 6 hours at 37°C in the presence or absence of A1 (50 μM). At the end of incubation period, the cells were collected using Accutase®, washed with ice-cold PBS, then stained for Annexin-V according to the manufacturer’s instructions prior to analysis using BD FACS Diva on CANTO II.

#### Evaluating ROS production

Production of ROS in MSCs was evaluated by DHE staining. Briefly, in a 12-well plate, 25 x 10^3^ cells were seeded per well. After 24 hours, the cells used as a positive control were treated with 2.5 μM of Dp44mT ROS-inducing agent, for 24 hours. Correspondent wells were treated with 50 μM A1 in the presence or absence of 1 mg/ml of OVA for 6 hours. The negative control was treated for 1 hour with 20 mM of NAC, a general cysteine donor. After incubation, the cells were washed with PBS, collected with trypsin, washed with ice-cold 2% FBS in PBS solution, then stained with DHE 10 μM (diluted in PBS) for 30 minutes at 37°C. After staining, cells were washed once with ice-cold2% FBS in PBS solution. The stained cells were resuspended in 2% FBS in PBS solution and kept on ice in the dark to be analyzed by BD FACS Diva on CANTO II within 1 hour.

#### Turbidity assay

To assess the level of aggregate formation a turbidity assay was used were OVA solution and A1 were admixed in serum-free AMEM to obtain 1mg /ml OVA and 50 μM A1 mixture. The turbidity was then evaluated vs AMEM alone, 1mg/ml OVA alone or 50μM A1 alone by transferring 100μL of each sample to a polystyrene flat bottom 96-well plate in 6 replicates. The wavelength for measurement was defined according to the examination of the absorbance spectra of the buffer (serum-free AMEM), in which no significant peak was observed. Thus, turbidity was assessed at 420 nm using a Synergy H1 microplate reader (BioTek). The plates were incubated at 37°C and shaken for 5 seconds before each reading. Absorbance was measured every 15 minutes. The experiment was conducted 4 times with 6 technical replicates for each condition.

#### Cytokine and chemokine analysis

To assess the profile of cytokine and chemokine production, 15 cm cell culture dishes containing MSCs at 70% confluency were grown in serum-free AMEM for 24 hours at 37°C and 5% CO_2_. MSCs were then treated with 50 μM of A1 in serum-free AMEM for 6 hours. The post-treatment supernatant was collected and kept at 4°C, and fresh serum-free AMEM was replenished without A1. After 24 hours of the initial A1 treatment, the supernatant was collected and added to the previous collection. All collected supernatant was combined and concentrated 80x using the Amicon Ultra-4 centrifugal filters (3000 NMWL) for 1 hour at 4°C at 4500 x g. Collected concentrates were then aliquoted and frozen at -80°C until shipped to EveTechnologies (Calgary, AB, Canada) for cytokine/chemokine assessment by Luminex.

#### Generation of B16 tumor lysate

To prepare cell lysates, cultured B16 cells were collected using 0.05% trypsin then washed 3 times with PBS in centrifugation cycles of 1000 rpm for 10 min to remove traces of FBS. Washed cells were kept as a pellet at -80°C until lysis. For the lysis procedure, the cell pellet was subjected to 5 cycles if freezing in liquid nitrogen followed by thawing (at 37°C) cycles, with complete homogenization with vortex/shaking conducted before every freezing/thawing step. The final solution was centrifuged for 10 min at 4500 x g at 4°C and the protein lysate supernatant was collected, quantified, aliquoted and stored at -80°C until further use. Protein quantification was performed using Bio-Rad Protein Assay (Bio-Rad) according to manufacturer instructions.

### Quantification and statistical analysis

#### RNA-seq and Bioinformatic analysis

For RNA-seq, MSCs were treated with 50 μM A1 alone or 50 μM A1 + 1 mg/ml OVA for 6 hours. At the end of treatment period, the cells were detached, washed, and collected to extract their RNA using the RNeasy Mini Kit (QIAGEN). Quantification of total RNA was made by QuBit (ABI), and 500 ng of total RNA was used for library preparation. The quality of total RNA was assessed with the BioAnalyzer Nano (Agilent), and all samples had a RIN above 8. Library preparation was done with the KAPA mRNAseq stranded kit (KAPA, Cat no. KK8420). Ligation was made with 9 nM final concentration of Illumina index, and 10 PCR cycles were required to amplify cDNA libraries. Libraries were quantified by QuBit and BioAnalyzer. All libraries were diluted to 10 nM and normalized by qPCR using the KAPA library quantification kit (KAPA; Cat no. KK4973). Libraries were pooled to equimolar concentration. Sequencing was performed with the Illumina Hiseq2000 using the Hiseq Reagent Kit v3 (200 cycles, paired-end) using 1.7 nM of the pooled library. All Fastq files (strand-specific sequencing, N=4 per group) were aligned to GRCm38 (mouse genome Ensembl release 102) with STAR (v2.7). Raw reads mapping to genomic features (summarized per gene) were extracted with featureCounts (strand-specific option). Expression matrices were filtered, genes with very low counts were removed, and protein-coding genes were kept for further analyses. Gene expression in both Accum-A1- and A1 + OVA-treated MSCs was compared to BM-derived MSC controls with DESeq2 to generate a ranked list of differentially expressed genes based on the log2 fold change. Gene set enrichment on either ranked lists of genes or a number of significantly up-or down-unregulated genes perturbed by A1 alone or admixed with Accum A1 variant compared to MSC controls were performed using the Reactome collection of pathways. The variance stabilizing transformation was applied to gene expression matrices prior to visualization. If not mentioned in the text, the significance threshold is set to 5% after p-value adjustment with the Benjamini–Hochberg method to control for false positives among differentially expressed genes (DEGs). All custom scripts, including the prediction of putative targets, were written in R programming and statistical language. Data visualization was made with ggplot2, enrichplot, Upset plots, and Pheatmap R functions.

#### Quantification and statistical analysis

*p-*values were calculated using one-way analysis of variance (ANOVA) or Log-rank test for animal survival experiments. Results are represented as average mean with standard deviation (S.D.) error bars, and statistical significance is represented with asterisks: ∗*p* ˂ 0.05, ∗∗*p* ˂ 0.01, ∗∗∗*p* ˂ 0.001.
