# Supplementary material for: A1-reprogrammed mesenchymal stromal cells prime potent antitumoral responses
Source: iScience. 2024 Feb 17;27(3):109248. doi: 10.1016/j.isci.2024.109248 (PMC10907831; doi:10.1016/j.isci.2024.109248)
Supplement: Document S1. Figures S1–S7 [file mmc1.pdf]

iScience, Volume *27*

Supplemental information

A1-reprogrammed mesenchymal stromal cells prime potent antitumoral responses

Marina Pereira Gonçalves, Roudy Farah, Jean-Pierre Bikorimana, Jamilah Abusarah, Nehme EL-Hachem, Wael Saad, Sebastien Talbot, Daniela Stanga, Simon Beaudoin, Sebastien Plouffe, and Moutih Rafei

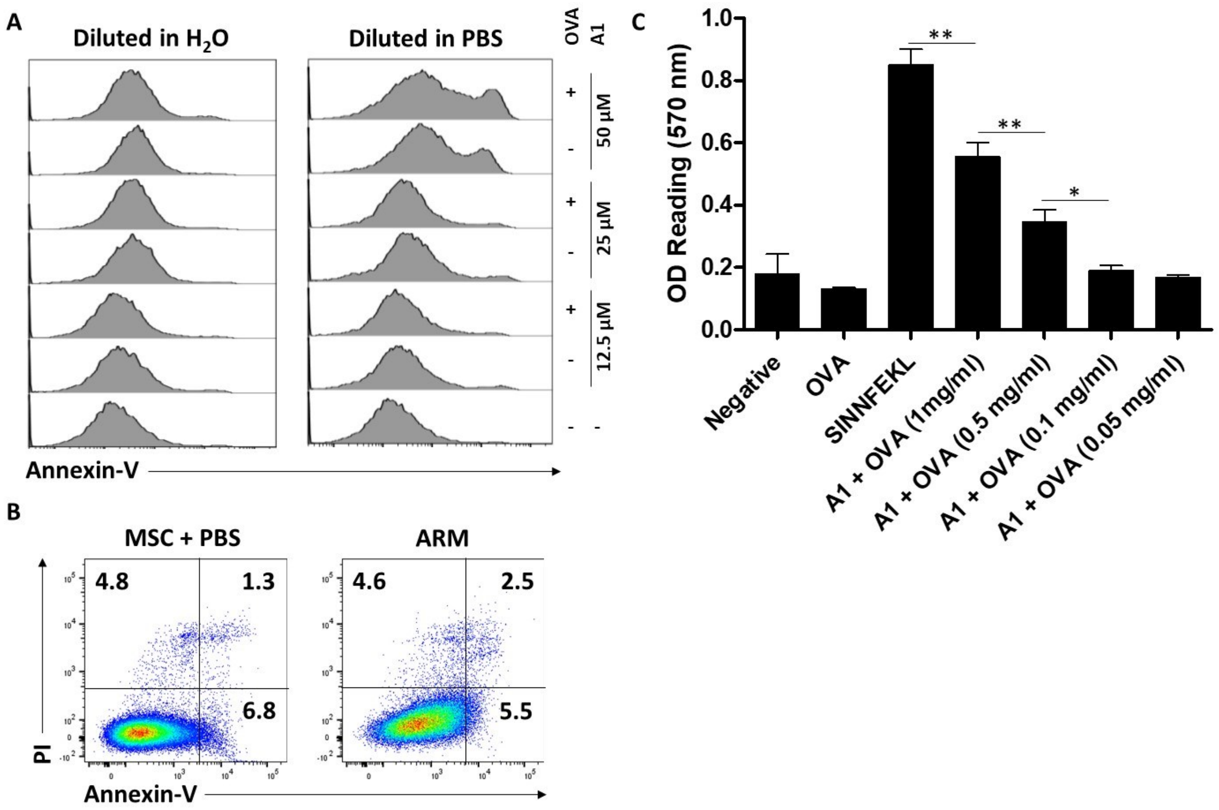

Figure S1, related to Figure 2. Optimizing the A1 treatment conditions to tune cross-presentation response induction in MSCs. A) Flow-cytometry assessment of apoptosis in response to ascending A1 doses diluted in water versus PBS. **B)** Apoptosis/PI co-staining of control MSCs (left side) versus MSCs treated with OVA mixed with A1 at 25 μM (right side). **C)** Antigen cross-presentation assay conducted using different OVA concentrations to find the minimal concentration needed to induce cross-presentation in MSCs.

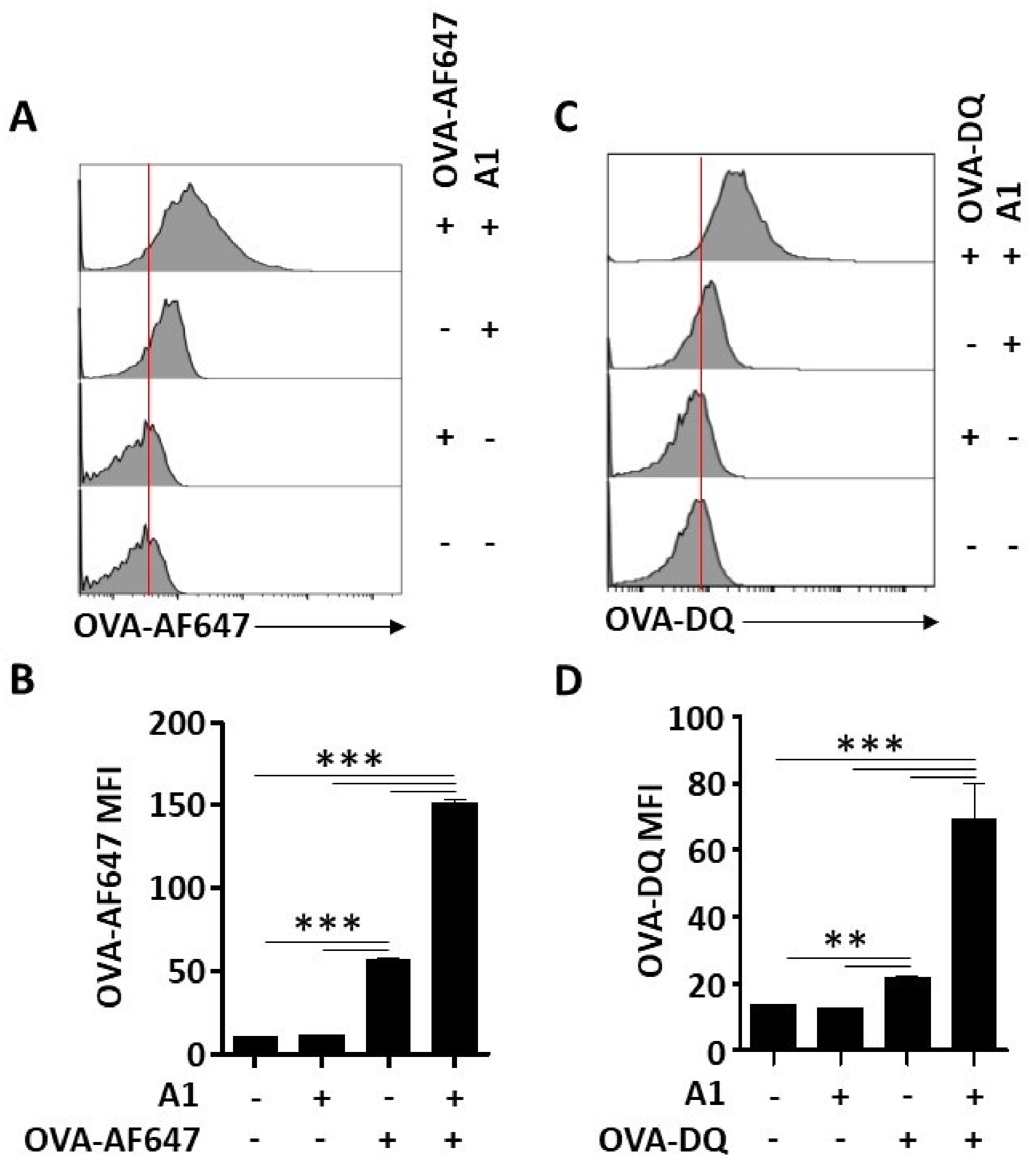

Figure S2, related to Figure 2. Validating the antigen cross-presentation properties of A1 on human MSCs. A) Representative flow-cytometry analysis of OVA uptake by A1-treated human MSCs. **B)** Signal quantification of the results presented in panel A. **C)** Representative flow-cytometry analysis of OVA processing by A1-treated human MSCs. **D)** Signal quantification of the results presented in panel C.

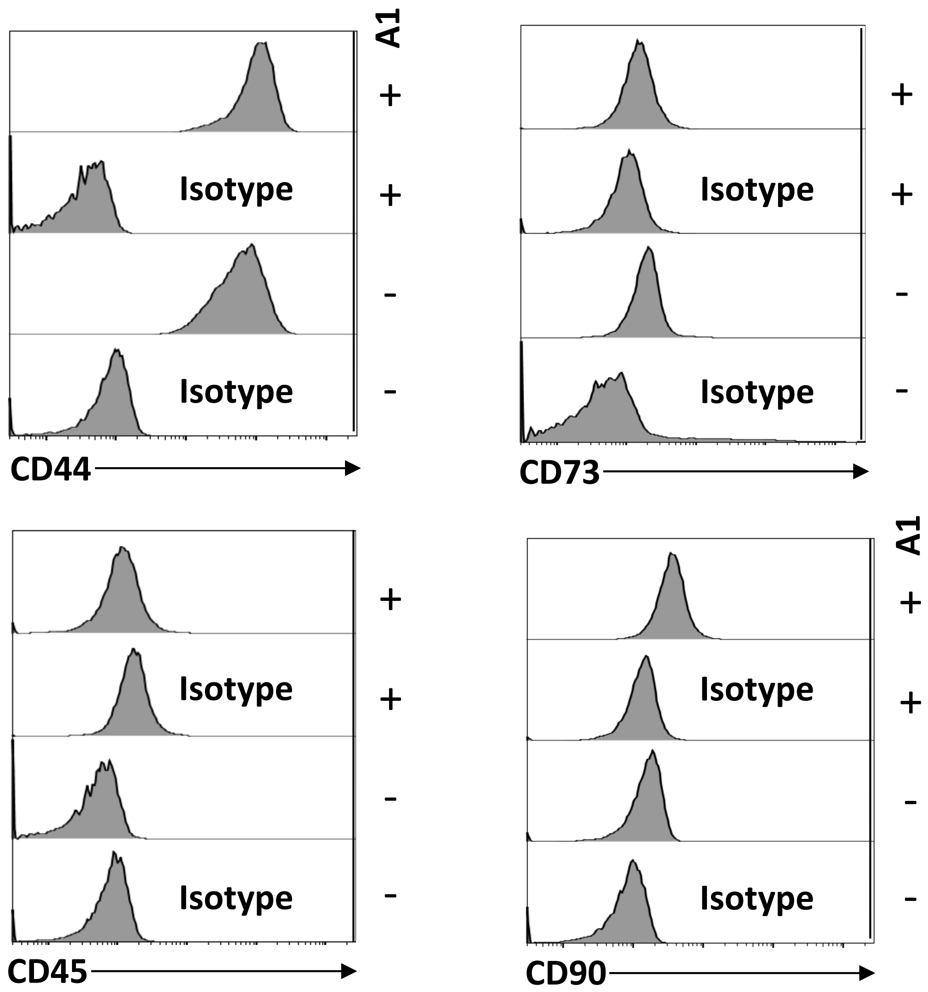

Figure S3, related to Figure 2. Phenotype characterization of A1-treated MSCs. Control versus A1-treated MSCs were stained by flow cytometry to assess the expression of CD44, CD45, CD73, and CD90. For each surface marker, from the bottom to the top, the first and third rows comprise the isotype, and the second and forth row comprise the targeted surface marker.

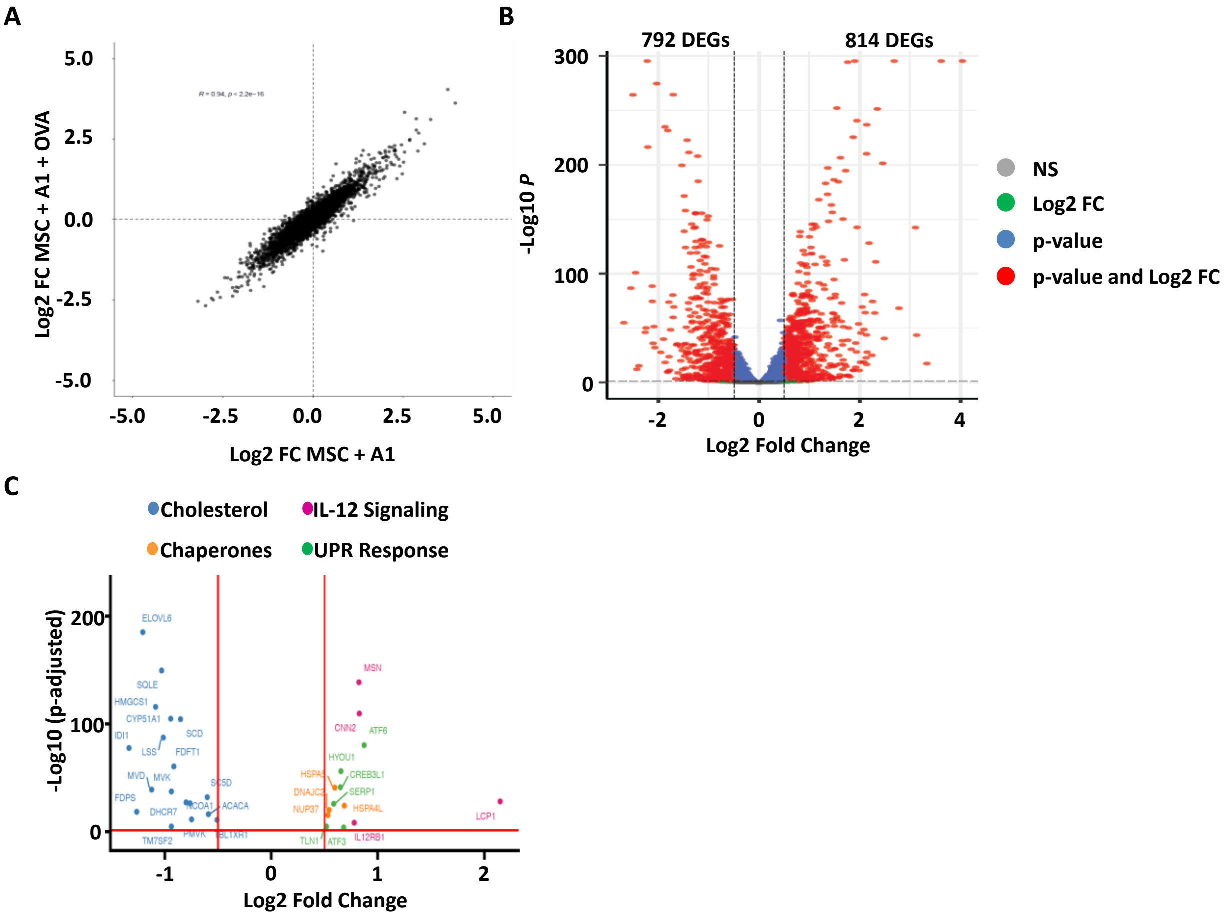

Figure S4, related to Figure 4. Characterizing the molecular response of A1-treated MSCs. **A)** Correlation plot showing Spearman's rank correlation coefficient of DEGs (log2 fold changes) shows the significant similarity of gene expression patterns between the A1 and A1+ OVA groups. **B)** Volcano plot representing differentially expressed genes in response to A1. **C)** Volcano plot depicting some important biological processes modulated in MSCs in response to A1. All genes from corresponding reactome analyses and showing a log2FC greater or equal to 0.5 are labeled for further investigation.

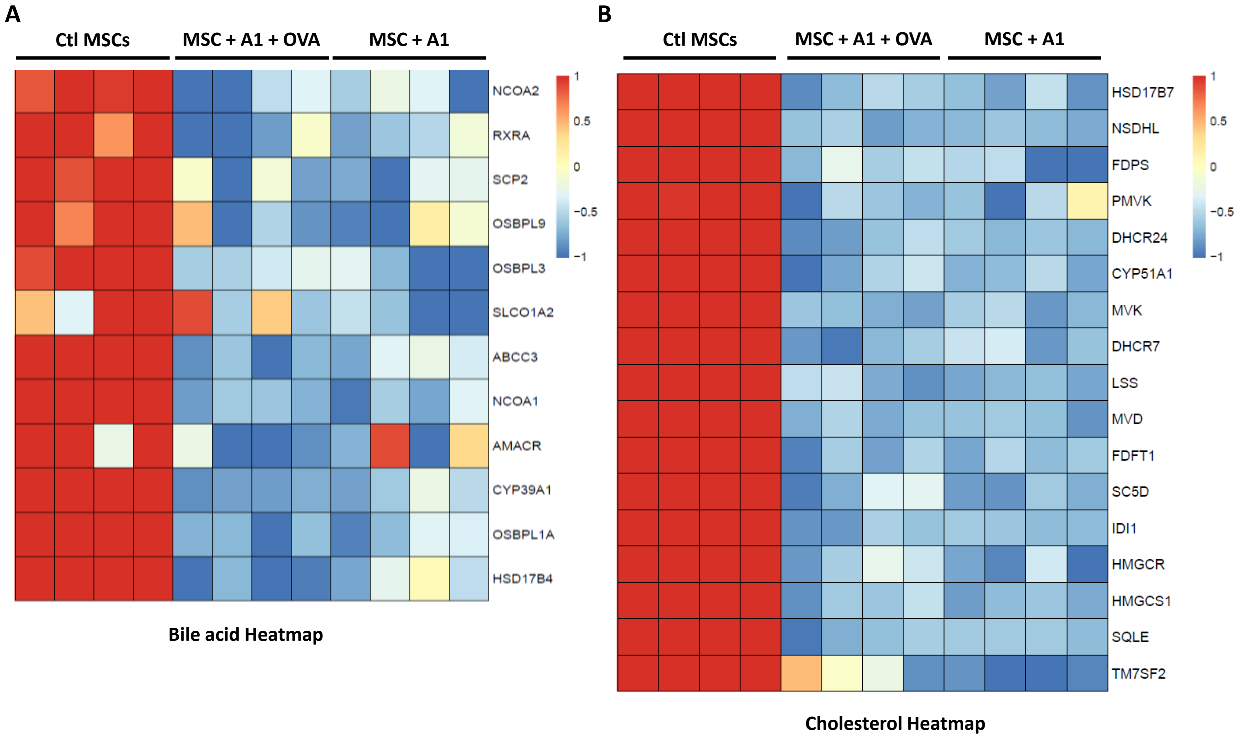

Figure S5, related to Figure 4 Characterizing the molecular response of A1-treated MSCs (continued). A) Heatmap depicting genes related to bile acid metabolism that are downregulated by A1 treatment. **B)** Same as in A, except that represents genes involved in cholesterol biosynthesis. Genes showed in heatmaps A and B were also contributing to significant statistics from both differential expression and pathway analyses (FDR < 5%).

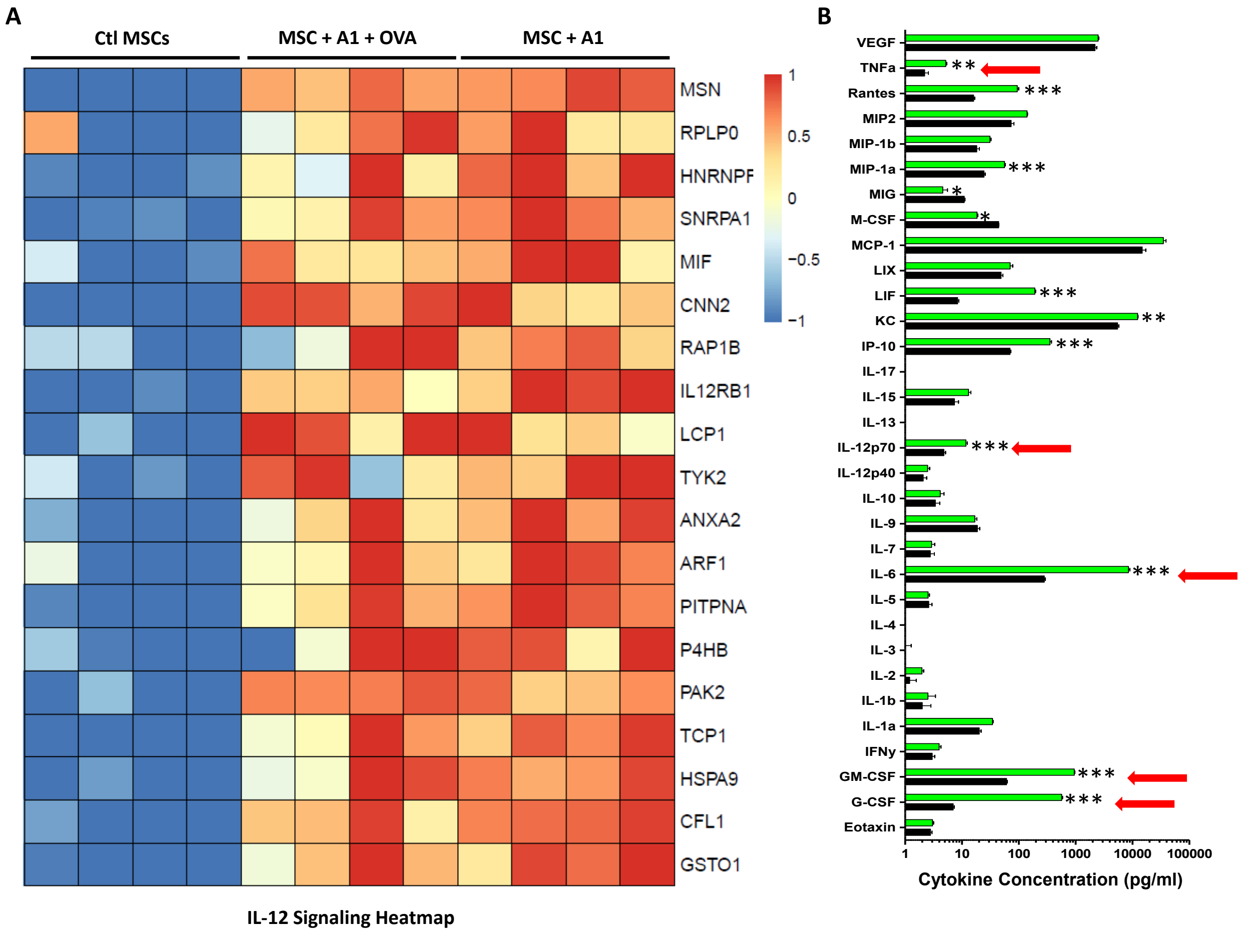

Figure S6, related to Figure 4. Characterizing the molecular response of A1-treated MSCs (continued). A) The IL-12 response heatmap depicting genes that are modulated by A1 treatment. The same description is in Supplementary Figure 4, and gene expression is scaled from -1 to 1 range. **B)** Luminex analysis of various cytokines in response to A1 treatment (in green). The red arrows highlighted pro-inflammatory cytokines of interest with significant changes. For this panel, n=6/group.

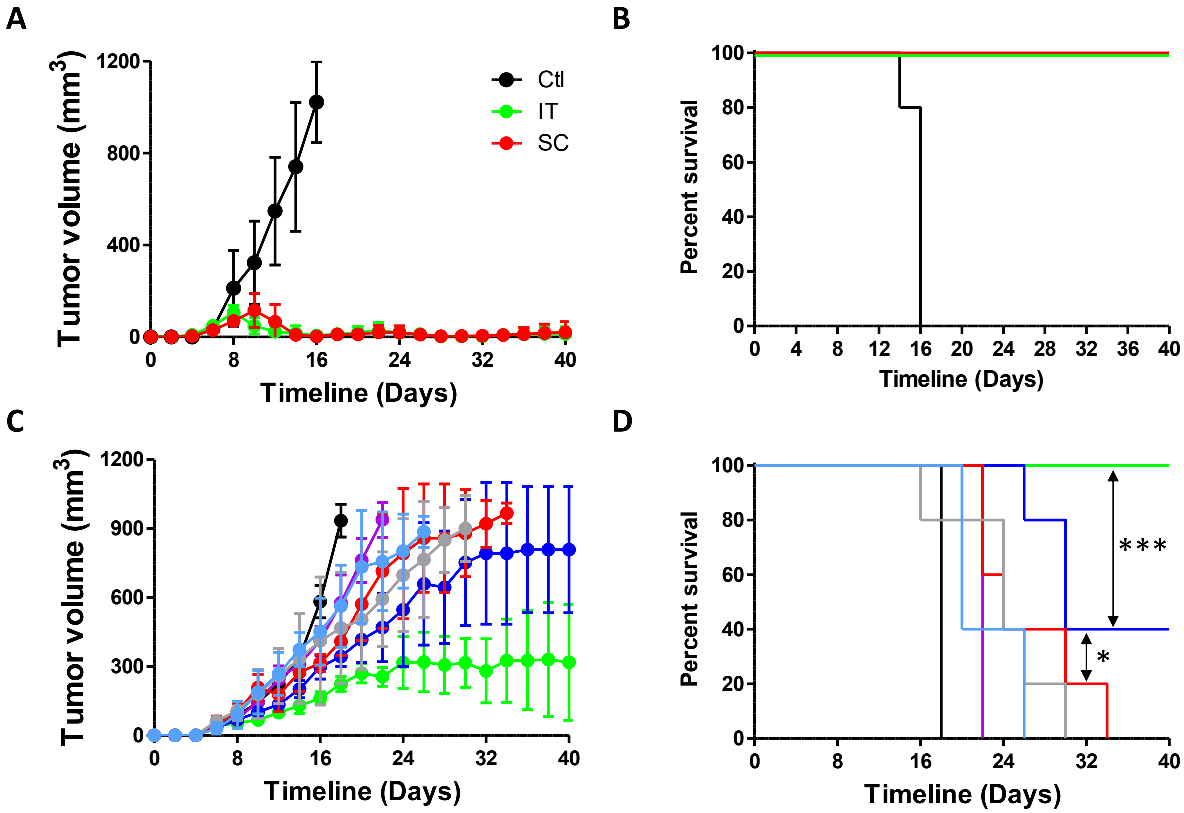

Figure S7, related to Figure 5. Comparing the routing and dosing of the allogeneic ARM vaccine. A) EG.7 tumor growth in response to IT or SC allogeneic ARM vaccination pulsed with OVA and co-delivered with anti-PD-1. The black line represents EG.7 growth (control group). **B)** Kaplan-Meier survival curve of the experiment shown in panel A. **C**) EG.7 tumor growth in response to OVA-pulsed allogeneic ARM delivered at different doses. The black line represents EG.7 growth (control group). As for the doses: Green (5×10^5), dark blue (2.5×10^5), red (1×10^5), gray (5×10^4), light blue (1×10^4), purple (5 x 103). **D)** Kaplan-Meier survival curve of the experiment shown in panel C. For this experiment, n=5/group.
